# Modelling the age-prevalence relationship in schistosomiasis: A secondary data analysis of school-aged-children in Mangochi District, Lake Malawi

**DOI:** 10.1016/j.parepi.2023.e00303

**Published:** 2023-05-03

**Authors:** Amber L. Reed, Angus M. O'Ferrall, Sekeleghe A. Kayuni, Hamish Baxter, Michelle C. Stanton, J. Russell Stothard, Christopher Jewell

**Affiliations:** a Lancaster Medical School, Lancaster University, Bailrigg House, Bailrigg, Lancaster LA1 4YE, UK; b Tropical Disease Biology, Liverpool School of Tropical Medicine, Pembroke PI, Liverpool L3 5QA, UK; c MASM Medi Clinics Limited, Medical Aid Society of Malawi (MASM), P.O. Box 31659, Lilongwe 3. Malawi; d Vector Biology, Liverpool School of Tropical Medicine, Pembroke Pl, Liverpool L3 5QA, UK; e Mathematics and Statistics, Lancaster University, Bailrigg House, Bailrigg, Lancaster LA1 4YE, UK

**Keywords:** *Schistosoma mansoni*, *Schistosoma haematobium*, Co-infection, Generalised additive models, Age profiling, School-aged-children

## Abstract

Schistosomiasis is an aquatic snail borne parasitic disease, with intestinal schistosomiasis (IS) and urogenital schistosomiasis (UGS) caused by *Schistosoma mansoni* and *S. haematobium* infections, respectively. School-aged-children (SAC) are a known vulnerable group and can also suffer from co-infections. Along the shoreline of Lake Malawi a newly emerging outbreak of IS is occurring with increasing UGS co-infection rates. Age-prevalence (co)infection profiles are not fully understood. To shed light on these (co)infection trends by *Schistosoma* species and by age of child, we conducted a secondary data analysis of primary epidemiological data collected from SAC in Mangochi District, Lake Malawi, as published previously. Available diagnostic data by child, were converted into binary response infection profiles for 520 children, aged 6–15, across 12 sampled schools. Generalised additive models were then fitted to mono- and dual-infections. These were used to identify consistent population trends, finding the prevalence of IS significantly increased [*p* = 8.45e-4] up to 11 years of age then decreasing thereafter. A similar age-prevalence association was observed for co-infection [*p* = 7.81e-3]. By contrast, no clear age-infection pattern for UGS was found [*p* = 0.114]. Peak prevalence of *Schistosoma* infection typically occurs around adolescence; however, in this newly established IS outbreak with rising prevalence of UGS co-infections, the peak appears to occur earlier, around the age of 11 years. As the outbreak of IS fulminates, further temporal analysis of the age-relationship with *Schistosoma* infection is justified. This should refer to age-prevalence models which could better reveal newly emerging transmission trends and *Schistosoma* species dynamics. Dynamical modelling of infections, alongside malacological niche mapping, should be considered to guide future primary data collection and intervention programmes.

## 1 Introduction

School-aged-children (SAC) are known to be one of the most vulnerable groups for schistosomiasis, which can lead to severe morbidity, and in some cases mortality. Standard infection and transmission rates in SAC are 3–4 times higher than in adults (Colley et al., 2014). Children are thought to be first infected soon after birth upon freshwater contact(s) with prevalence increasing with cumulative parasite exposure(s) up to adolescence (WHO, 2022). Over time, ongoing inflammation within the tissues, from accumulating trapped eggs, can lead SAC to suffer from malnutrition, anaemia, and neurological and developmental delays (Mawa et al., 2021). Furthermore, acute and chronic infection with urogenital schistosomiasis (UGS) and/or intestinal schistosomiasis (IS) can lead to debilitating symptoms and signs such as stunting, but whether chronic co-infections are truly synergistic is equivocal (Gouvras et al., 2013).

To counter schistosomiasis, WHO recommend preventive chemotherapy by mass drug administration (MDA) with the anthelmintic praziquantel. MDA treatment programmes can avert and reverse some of these disease manifestations as well as diminish transmission. However, praziquantel is only effective against adult worms, leaving immature (drug tolerant) worms to remain within the body (Colley et al., 2014). Since MDA does not guard against reinfection, SAC often reacquire infection upon subsequent water contact, with persistent “hotspots” occurring (Mawa et al., 2021; Stothard et al., 2013; Makaula et al., 2014; McManus et al., 2018). As a consequence of ongoing persistent schistosomiasis infection among SAC, children are often absent from school, and have delayed learning affecting their ability to work as they enter adulthood (WHO, 2022). This further hinders the socio-economic advances of a geographical area, a known risk factor for schistosomiasis (Mawa et al., 2021).

A decrease in prevalence of infection is known to occur after young adolescence, which is typical of community age-prevalence relationships (WHO, 2022; Woolhouse, 1998). This is thought to be due to the development of partial immunity over time given repeated exposure, as well as decreased contact with water or more enigmatic changes in skin texture, for example (Colley et al., 2014; Dawaki et al., 2016; Oso and Odaibo, 2020). Prevalence rates among SAC and the wider population vary considerably between geographical areas, often with localised rates among each community (Colley et al., 2014). There are many factors that influence the transmission rates in a specific area, such as demographic and environmental factors, MDA, and snail-schistosome ecology (Mawa et al., 2021). Consequently, prevalence data can be noisy but pooling across schools allows for inferences to be extracted. The heterogeneity of transmission in a geographical area within a community influences the age at which prevalence and intensity of infection are at their highest in SAC, leading to some SAC being burdened more than others (Gryseels et al., 2006). The identification of areas with high prevalence and intensity of infection is essential to allow for more appropriate application of control interventions, such as MDA (Kittur et al., 2017).

The southern part of the Lake Malawi shoreline in Mangochi District has been reported to have increasing schistosomiasis infection rates since the 1980s, with known UGS endemicity in the region (Madsen et al., 2011). Al-Harbi et al. 2019 (Alharbi et al., 2019) and Kayuni et al. 2020 (Kayuni et al., 2020) reported the emergence and an outbreak of IS since 2017 in this region, in part due to the newly detected presence of *Biomphalaria*, a keystone snail intermediate host for *Schistosoma mansoni*. They suggested better inspection of age-infection dynamics is needed before intensification of current control methods is advised. Similarly, with the recent endorsement of urine-CCA testing for prevalence mapping of IS (Bärenbold et al., 2018), closer inspection of infection data by age would further underpin its guiding role. To our knowledge, however, there are no studies that have analysed the age-prevalence relationship of IS within SAC in the context of a newly emerging focus of infection set against a background of UGS.

In this secondary analysis of primary data reported by Kayuni et al. 2020 (Kayuni et al., 2020), our two aims were: i) to determine if general relationships between age of SAC and prevalence of IS, UGS and co-infection could be determined, and ii) to assess heterogeneities in infection-age profiles across sampled schools.

## 2 Methods

### 2.1 Dataset

**Fig. 1:**
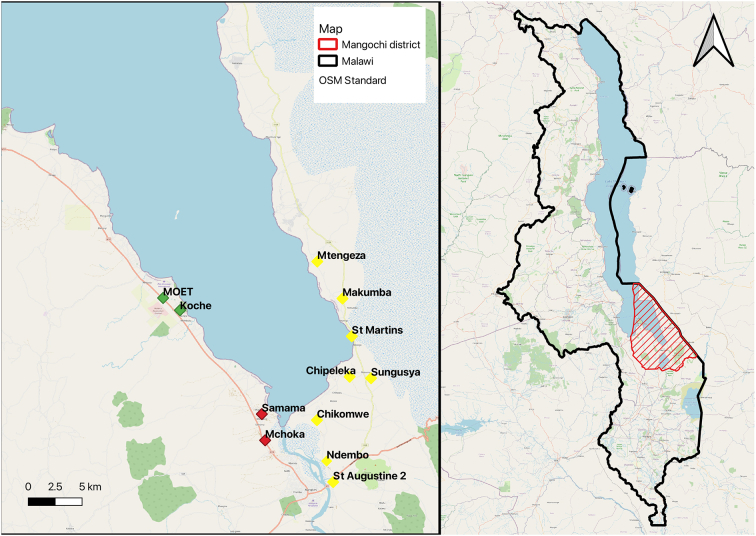
Locations of the schools sampled in the primary study, a) red markers represent a repeat of the previous collection (80 SAC sampled), green markers represent collections newly known to *Biomphalaria* intermediate host locations (60 SAC sampled) and yellow markers represent rapid mapping of the shoreline (30 SAC sampled), b) map indicating the location of Mangochi District. (For interpretation of the references to colour in this figure legend, the reader is referred to the web version of this article.)

The primary dataset reported by Kayuni et al. (Kayuni et al., 2020) which this secondary analysis is based on, was originally collected in late May/June 2019 from cross-sectional school-based surveys in Mangochi District along the shoreline of southern Malawi (Fig. 1) (Kayuni et al., 2020). In brief, the study carried out a mixture of rapid diagnostic tests, parasitological examinations and questionnaire surveys on 520 primary school children, aged 6–15 years old in twelve schools, after parental consent was given. The study was split into three phases during May/June 2019: 80 SAC each from Samama and Mchoka schools – annual follow up (Alharbi et al., 2019); 60 SAC each from Moet and Koche schools – an assessment of the two schools near known locations for *Biomphalaria*; and 30 SAC each from 8 further schools along the lake shoreline – a rapid surveillance exercise. The SAC were randomly sampled after being stratified by gender and age, with sample sizes at each school calculated by standard sample size methodology (Kayuni et al., 2020). As reported by Kayuni et al. (Kayuni et al., 2020), all participants provided a urine sample. Sampling was accompanied by a questionnaire on demographics, water contact behaviour, praziquantel treatment history and travel. A visual inspection of the urine samples was carried out before samples underwent on-site testing using the circulating cathodic antigen (CCA) test for IS, and 10 ml well-mixed urine was filtrated for UGS (Kayuni et al., 2020). The former was used to estimate prevalence of IS and the latter for UGS (Bärenbold et al., 2018; WHO, 1993).

Ethical approval for this study was obtained from the National Health Sciences Research Committee, Mangochi District Health Office Research Committee and LSTM's Research Ethics Committee.

### 2.2 Statistical analysis

The primary data were cross-checked with any ambiguities resolved against paper records, then secondary analyses were carried out in R version 3.6.1 with RStudio. The CCA antigen test and urine filtration results were used as binary response variables to measure the prevalence of infection. The responses were categorised into two subgroups ‘1 = Positive’ and ‘0 = Negative’ in our study. For CCA antigen tests in the original study an additional ‘trace’ result was recorded. In our study, we carry out two analyses: one as ‘T+’ (Trace positive) and one as ‘T-’ (Trace negative). ‘T+’ is where all trace responses are considered ‘Positive’ and ‘T-’ is where all trace responses are considered ‘Negative’.

As a visual exploration tool, heatmaps were used to inspect the empirical age-prevalence profile of *S. mansoni* and *S. haematobium* in each school. The order of the schools on the heatmaps reflected a highest to lowest prevalence ranking.

For both *Schistosoma* species assessed, our response data were binary: an individual was denoted positive (1) or negative (0) for infection and for co-infection an individual was positive (1 and 1) for both infections. Our response data were from independent tests detecting different infections. We assumed therefore, given the characteristics of a child, their age and school, that test results are independent between children. The CCA and urine filtration tests behave the same with respect to school and age, but sensitivity and specificity can vary with prevalence. Despite this, we assumed that the sensitivity and specificity do not change with respect to age of the children or by school.

We assumed the diagnostic data followed a Bernoulli distribution and therefore used a logistic regression framework. Since our exploratory data analysis (Appendix A Figs. A.1 and A.2) suggested a non-linear relationship between log odds of infection and age, we fitted age using a thin plate spline. School was additionally fitted as a categorical explanatory variable to adjust for systematic school-level variation in baseline prevalence. The resulting logistic generalised additive model (GAM) enables estimation of a smooth, though non-linear, relationship between age and prevalence as a trend summary of our otherwise noisy observational data (Hastie and Tibsh, 1990). GAMs were fitted using the ‘mgcv’ package in R version 3.6.1 (Wood, 2019) (Appendix B). After fitting these models, smooth age-prevalence curves were reconstructed for each outcome. Model fit was assessed by plotting the average of binned residuals against the fitted values as shown in Appendix E (Gelman and Hill, 2007).

## 3 Results

As reported in the primary study, 520 children were tested using urine CCA*-*dipsticks for *S. mansoni* and urine filtration for *S. haematobium (*Kayuni, 2020*)*. Our provisional secondary analysis found that the prevalence of *S. mansoni* at each school ranged from 67.5% to 96.7%, with overall pooled prevalence of 82.5% [T+]. *Schistosoma haematobium* prevalence ranged from 3.3% to 60.0% with an overall pooled prevalence of 24.0%. Co-infection prevalence by school, ranged from 1.67% to 56.7% with overall pooled prevalence of 21.0% (Table 1). Ages of the SAC were between 6 and 15 years, with mean age 10.4. Ndembo school had the lowest mean age sampled with 9.77, whereas Mtengza had the highest with 10.7. Trace negative [T-] prevalence summary can be found in Appendix Table D.1.

**Table 1:** Summary of prevalence of *S. mansoni* [T+]*, S. haematobium* and co-infection [T+].

	*S. mansoni* (CCA)[T+]		*S. haematobium* (Urine filtration)		Co-infection [T+]	
Name	No. Positive (%)	95% CI	No. Positive (%)	95% CI	No. Positive (%)	95% Cl
Mchoka (*N* = 80)	54 (67.5)	57.5–77.5	15 (18.9)	10.0–27.5	11 (13.8)	6.25–21.3
Samama (N = 80)	65 (81.3)	72.5–88.8	45 (56.2)	45.0–67.5	38 (47.5)	36.3–58.8
MOET (N = 60)	49 (81.7)	71.7–90.0	5 (8.33)	1.70–15.0	4 (6.67)	1.67–13.3
Koche (N = 60)	54 (90.0)	81.7–90.0	1 (1.67)	0.00–5.00	1 (1.67)	0.00–5.00
St Augustine 2 (N = 30)	29 (96.7)	90.0–100	13 (43.3)	26.7–60.0	12 (40.0)	23.3–56.7
Ndembo (N = 30)	25 (83.3)	70.0–96.7	18 (60.0)	43.3–76.7	17 (56.7)	40.0–73.3
Sungusya (N = 30)	27 (90.0)	76.7–100	5 (16.7)	3.33–30.0	4 (13.3)	3.33–26.6
St Martins (N = 30)	27 (90.0)	80.0–100	1 (3.33)	0.00–10.0	1 (3.33)	0.00–10.0
Chikomwe (N = 30)	24 (80.0)	63.3–93.3	3 (10.0)	0.0–23.3	3 (10.0)	0.00–23.3
Chipeleka (N = 30)	27 (90.0)	76.7–100	8 (26.7)	13.3–43.3	7 (23.3)	10.0–40.0
Makumba (N = 30)	23 (76.7)	60.0–90.0	2 (6.67)	0.00–16.7	2 (6.67)	0.00–16.7
Mtengeza (N = 30)	25 (83.3)	70.0–96.7	9 (30.0)	13.3–46.7	9 (30.0)	13.3–46.7
Total (N = 520)	429 (82.5)	79.2–85.8	125 (24.0)	20.4–27.7	109 (21.0)	17.5–24.4

### 3.1 Prevalence heatmaps

**Fig. 2:**
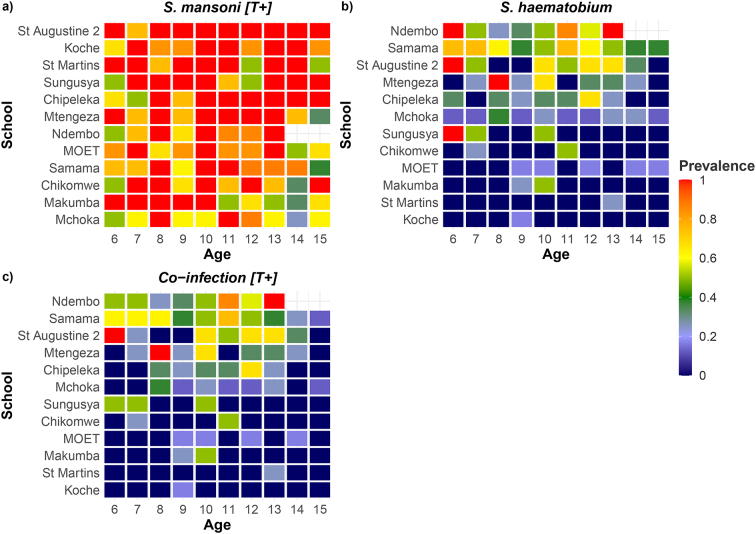
Heatmap showing the age of the children vs school prevalence for a) *S. mansoni* [T+] b) *S. haematobium* c) co-infection [T+]. Order of schools on heatmap was by highest to lowest prevalence and showed that there was considerable heterogeneity between the schools. Further*, S. haematobium* shows a similar pattern of prevalence among SAC to co-infection.

Fig. 2 shows that there was considerable heterogeneity between the schools. Further*, S. haematobium* shows a similar pattern of prevalence among SAC to co-infection. Trace negative result [T-] can be found in Appendix C Fig. C.1.

### 3.2 Generalised additive models

The thin-plate spline for age, adjusted for school, used in the GAM enables us to construct a smooth function of the log odds ratio of infection with respect to age. For the average binned residuals, no evidence of outliers or systematic model fit was found, suggesting they were a good measure of fit (Appendix E Fig. E.1 and E.2).

**Fig. 3:**
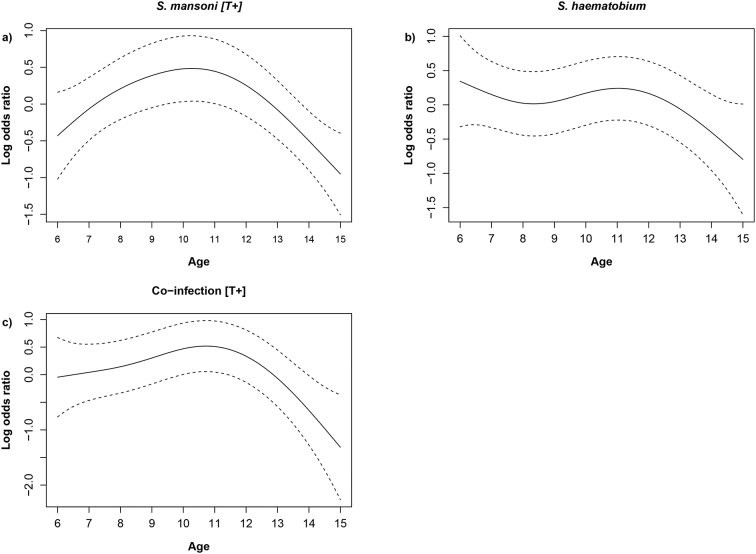
Thin plate spline functions of the log odds ratio of *Schistosoma* infection by age in SAC for (a) *S. mansoni* [T+] (b) *S. haematobium* (c) co-infection [T+]. This follows a general trend towards SAC between 9 and 12 years old having the highest odds of infection was seen in all cases, though that for UGS is not statistically significant.

Fig. 3a shows very strong evidence for a non-linear relationship between *S. mansoni* infection and age [T+: *p* = 8.45e-4], Fig. 3b shows strong evidence for a non-linear relationship between co-infection and age [T+: *p* = 7.81e-3], whereas there is no evidence to suggest an increase or decrease of *S. haematobium* infection with age [*p* = 0.114]. This is visualised in Fig. 3a, *S. mansoni* [T+], where the smoothing coefficient for age goes from a negative to positive from ages 6 to 11 before decreasing back to negative, and similarly for co-infection in Fig. 3b. For *S. haematobium* there was no clear pattern between prevalence and age for all the schools (Fig. 3c). *Schistosoma mansoni* [T-] and co-infection [T-] GAM adjusted for age and school result can be found in Appendix D Fig. D.1.

In comparison to Mchoka School (baseline), the log odds of being positive for *S. mansoni* infection increased by 0.767 [T+] per year [*p* = 4.40e-2, 95% CI: 2.06e-2, 1.51] at Samama. Similarly, the log odds of being positive for *S. mansoni* infection [T+] increased by 1.52 [*p* = 2.37e-3, 95% CI: 0.540, 2.50] at Koche, 2.63 [*p* = 1.21e-2, 95% CI: 0.576, 4.69] at St Augustine 2, 1.48 [*p* = 2.50e-2, 95% CI: 0.186, 2.78] at Sungusya, 1.47 [*p* = 2.67e-2, 95% CI: 0.169, 2.76] at St Martins, and 1.47 [*p* = 2.68e-2, 95% CI: 0.168, 2.76] at Chipelekera school per year (Table 2). For *S. haematobium,* the following significant coefficient estimates suggest that as a SAC ages, the log odds of being positive for *S. haematobium* infection decreases by −2.62 [*p* = 1.24e-2, 95% CI: −4.68, −0.657] at Koche, increases by 1.75 [*p* = 1.99e-6, 95% CI: 103, 2.47] at Samama, 1.19 [*p* = 1.12e-2, 95% CI: 0.271, 2.12] at St Augustine 2, and 1.74 [*p* = 2.52e-4, 95% CI: 0.807, 2.67] at Ndembo per year compared to Mchoka (Table 2). Trace negative [T-] result of the GAM can be found in Appendix Table D.2.

**Table 2:** Coefficients for the GAM with smooth term age adjusted for school.

	*S. mansoni* [T+]		*S. haematobium*		Co-infection [T+]	
		95% Cl		95% CI		95% CI
Smooth term (*p-value*)						
Age	8.45e-4***		0.114		7.81e-3**	
*Factor (*estimated *coefficient) School*						
Samama	0.767*	(0.0206,1.51)	1.75***	(1.03, 2.47)	1.81***	(1.01, 2.59)
MOET	0.796^**.**^	(−0.0251,1.62)	−0.940^**.**^	(−0.202,0.138)	−0.815	(−2.02, 0.391)
Koche	1.52**	(0.540, 2.50)	−2.62*	(−4.68, −0.567)	−2.26*	(−4.34, −0.180)
St Augustine 2	2.63*	(0.576, 4.69)	1.19*	(0.271, 2.12)	1.43**	(0.440, 2.41)
Ndembo	0.621	(−0.465, 1.71)	1.74***	(0.807, 2.67)	1.89***	(0.920, 2.86)
Sungusya	1.48*	(0.186, 2.78)	−0.143	(−1.26,0.975)	−4.33e-2	(−1.28, 1.20)
St Martins	1.47*	(0.169, 2.76)	−1.92^**.**^	(−3.99, 0.155)	-1.57	(−3.67, 0.532)
Chikomwe	0.634	(−0.393, 1.66)	−0.745	(−2.07, 0.578)	−0.387	(−1.75, 0.97)
Chipeleka	1.47*	(0.168,2.76)	0.445	(−0.546, 1.43)	0.628	(−0.444, 1.70)
Makumba	0.484	(−0.503, 1.47)	−1.16	(−2.70, 0.386)	−0.788	(−2.37, 0.791)
Mtengza	0.928^**.**^	(−0.160, 2.02)	0.656	(−0.314,1.63)	1.05*	(0.0228, 2.07)
Mchoka	0	–	0	–	0	–

*Significance *p* < 0.05, **Significance *p* < 0.01, ***Significance *p* < 0.001, Significance at *p* < 0.1.

For co-infection, the coefficient estimate for Samama school suggests a significant relationship with age such that the log odds of being positive for co-infection increases by 1.81 [*p* = 7.17e-6, 95% CI: 1.01, 2.59] [T+] per year compared to Mchoka. Similarly, the log odds of being positive for co-infection decreases by −2.26 [*p* = 3.32e-2, 95% CI: −4.34, −0.180] at Koche and increases by 1.43 [*p* = 4.58e-3, 95% CI: 0.440, 2.41] at St Augustine 2, 1.89 [*p* = 1.37e-4, 95% CI: 0.920, 2.86] at Ndembo and 1.05 [*p* = 4.51e-2, 95% CI: 2.28e-2, 2.07] at Mtengeza per year.

**Fig. 4:**
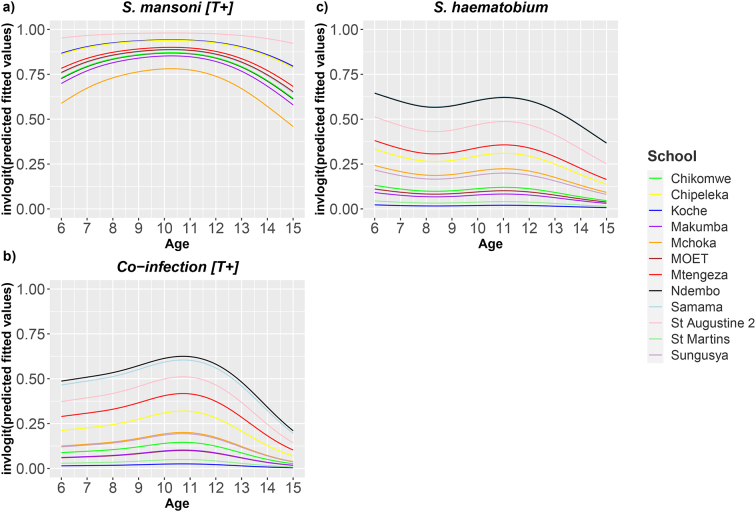
Smoothed age-specific prevalence of *Schistosoma* association with age of SAC for each school. a) *S. mansoni* [T+] b) *S. haematobium* C) co-infection [T+]. Light Green: Chikomwe, Yellow: Chipeleka, Dark Blue: Koche, Purple: Makumba, Orange: Mchoka, Brown: MOET, Red: Mtengeza, Black: Ndembo, Light Blue: Samama, Pink: St Augustine 2, Dark Green: St Martins, Mauve: Sungusya. (For interpretation of the references to colour in this figure legend, the reader is referred to the web version of this article.)

Given our fitted GAMs, Fig. 4 provides a prediction of the age-prevalence profile for each outcome in each school. The predictions indicate that prevalence was highly heterogeneous between schools, and highly heterogeneous in terms of the modelled infection outcome. The trace negative result [T-] can be found in Appendix Fig. D.2

## 4 Discussion

To our knowledge, the secondary analysis reported here is the first to analyse the IS infection age-relationship within SAC in a newly established and novel co-infection focus. The newly emerging focus of IS was first noted by Al-Harbi et al. (Alharbi et al., 2019) then described in greater detail by Kayuni et al. *(*Kayuni et al., 2020*).* Even though MDA has been ongoing, this focus of IS and co-infections thereof, is being further documented as it seemingly spreads along the southern part of shoreline of Lake Malawi in the Mangochi District. For *S. mansoni* infection detected by CCA dipsticks from the primary data (Kayuni et al., 2020), our secondary analysis finds that a positive association between IS prevalence and age was observed up to the age of 11, after which there was a decreasing trend [T+: *p* = 8.45e-4]. As might be expected, co-infection showed a similar pattern [T+: *p* = 7.81e-3], largely mirroring the IS pattern. By contrast, no clear age-infection pattern for UGS was identified [*p* = 0.114].

Other studies on *Schistosoma* infection carried out in sub-Saharan Africa have found varied peak infection profiles and all to be expected to arise around early to mid-adolescence [10–15 years] (Colley et al., 2014; Madsen et al., 2011; Singh and Muddasiru, 2014; Wilson, 2020; Mazigo et al., 2021). The earlier observed peak in *S. mansoni* and co-infection prevalence at the age of 11 in this study compared to up to 15 years may be a result of the newly established transmission potential of this species locally alongside growing acquired immunity in exposed children. This is due to their cumulative exposure to parasite antigens as adult worms die within the body after treatment or natural senescence, or as egg antigens present. Literature reports varied age infection profiles considered to be dependent on the transmission rates and focality (Woolhouse, 1998).

In classic infection epidemiology of schistosomiasis, changes in the ‘peak shift’ are known (Woolhouse, 1998; Anderson and May, 1985). These can be explained by site-specific factors, for instance, water exposure, environmental, socio-economic, genetic, MDA compliance, as well as age and gender profiles within a community (Mawa et al., 2021). In our instance, the expansion of the underlying distribution of *Bi. pfeifferi* both in time and space is an influential transmission potential driver of IS. A key observation is the contrasting age-prevalence by schistosome species, yet each share a common infection pathway, viz. exposure to unsafe water. The occurrence of the snail species present in unsafe water is an underlying heterogeneity of the fine-scale distributions of intermediate snail hosts, viz. *Biomphalaria* for *S. mansoni* and *Bulinus* for *S. haematobium*. The latter genus of snail is also undergoing a reappraisal as cryptic species, with as of yet unknown transmission potentials, as described in (Alharbi et al., 2022). Whilst Kayuni et al. 2020 (Kayuni et al., 2020) and Al-Harbi et al. 2019 (Alharbi et al., 2019) presented information of the presence and absence of *Biomphalaria*, a similarly precise map for *Bulinus* is starting to emerge (Alharbi et al., 2022). A recent study of *Bi. pfeifferi* has confirmed a year-on-year expanding distribution of this species along the shoreline of the lake, with clear evidence of schistosome DNA in examined snails from several independent locations (Alharbi et al., 2023). It is reasonable to speculate that further transmission foci for intestinal schistosomiasis will continue to appear in the lake and along its periphery.

Heterogeneities in prevalence among the schools were also found in our secondary analysis, with Mchoka School having the lowest and St Augustine 2 the highest for *S. mansoni* infection. Clearly, this heterogeneity indicates that there are many further un-considered factors that affect the transmission of *Schistosoma* infection within SAC, such as location, local environmental and socio-economic factors. For instance, SAC living and attending school in areas near the lake shoreline have been found to have increased and different age-infection profiles compared to inland villages (Madsen et al., 2011). As longitudinal data were not collected in the primary study, we were not able to assess seasonal or long-term variation in prevalence; however it is possible that the force of infection could vary spatially and temporally. For instance, environmental changes such as increases or decreases in water levels of the lake, flooding events or fluctuations in aquatic vegetation could impact SAC water contact (Alharbi et al., 2019; Kayuni et al., 2020). Another factor not studied was reinfection after preventive chemotherapy, as praziquantel only affects the adult worms, and any immediate snail exposure could therefore lead to reinfection. Further studies into the relationship between age, water exposure rates and treatment in the future could enhance our perspective of age infection profiles. Identifying the peak of infection prevalence within SAC at school level using GAM increases the interpretability of our findings by turning noisy data into useful and assessable information which in turn will help better our understanding of epidemiology of infection and other control methods along the southern part of the shoreline (Mwinzi et al., 2015).

A further limitation of this secondary analysis was that, owing to available resourcing, sample size taken from each school in the primary data was constrained (Kayuni et al., 2020). More generally, GAMs are sometimes known to smooth out underlying relationships excessively. Also, they have higher computational load compared to linear models and unstable behaviours at the boundaries of smooth splines (Laskowski et al., 2020). Nevertheless, for the purpose of our study, the general age-prevalence relationships were detected adequately, and provide a useful insight for future research into the causal mechanisms driving this infection biology.

## 5 Conclusion

Our study which is a secondary analysis of recently collected epidemiological data concerning a newly emergent focus of IS against an existing background of US, provides evidence for the peak of prevalence for *Schistosoma* infection being around 11 years for both *S. mansoni* mono-infection and co-infection with *S. haematobium* along the southern part of Lake Malawi in Mangochi district. However, considerable heterogeneity still remains in terms of baseline prevalence between schools, and investigating this in terms of demographics and *Schistosoma* transmission dynamics requires further research. In particular, understanding how SAC exposure is related to water access will require both further prevalence and malacological niche mapping. Coupling these conclusions into statistically-grounded infection modelling techniques will advance the understanding of the dynamics of *Schistosoma* infection, and hence inform future intervention programmes.

## Declaration of Competing Interest

None.

## Data Availability

Anonymised epidemiological data are available from the corresponding author upon request.

## Appendix A Supplementary data

Supplementary material Image 1
