# Supplementary material for: Modelling the age-prevalence relationship in schistosomiasis: A secondary data analysis of school-aged-children in Mangochi District, Lake Malawi
Source: Parasite Epidemiol Control. 2023 May 3;22:e00303. doi: 10.1016/j.parepi.2023.e00303 (PMC10205779; doi:10.1016/j.parepi.2023.e00303)
Supplement: Supplementary file 1 — Supplementary material [file mmc1.pdf]

Appendix

A. Raw data

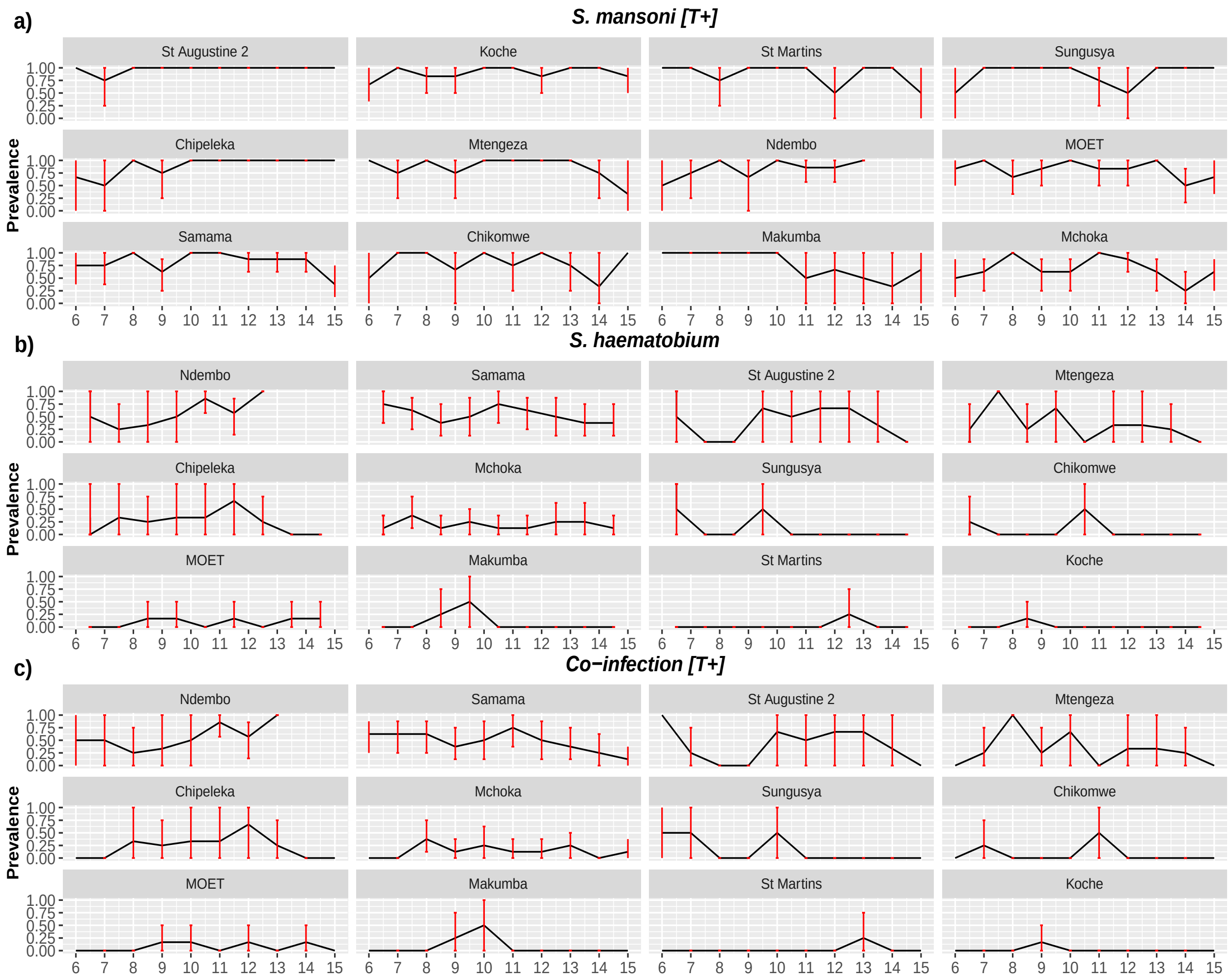

Figure A.1: Raw data plot showing the age of the children vs school prevalence for a) *S. mansoni* [T+], b) co-infection [T+] and c) *S. haematobium*. Order of schools on heatmap was by highest to lowest prevalence.

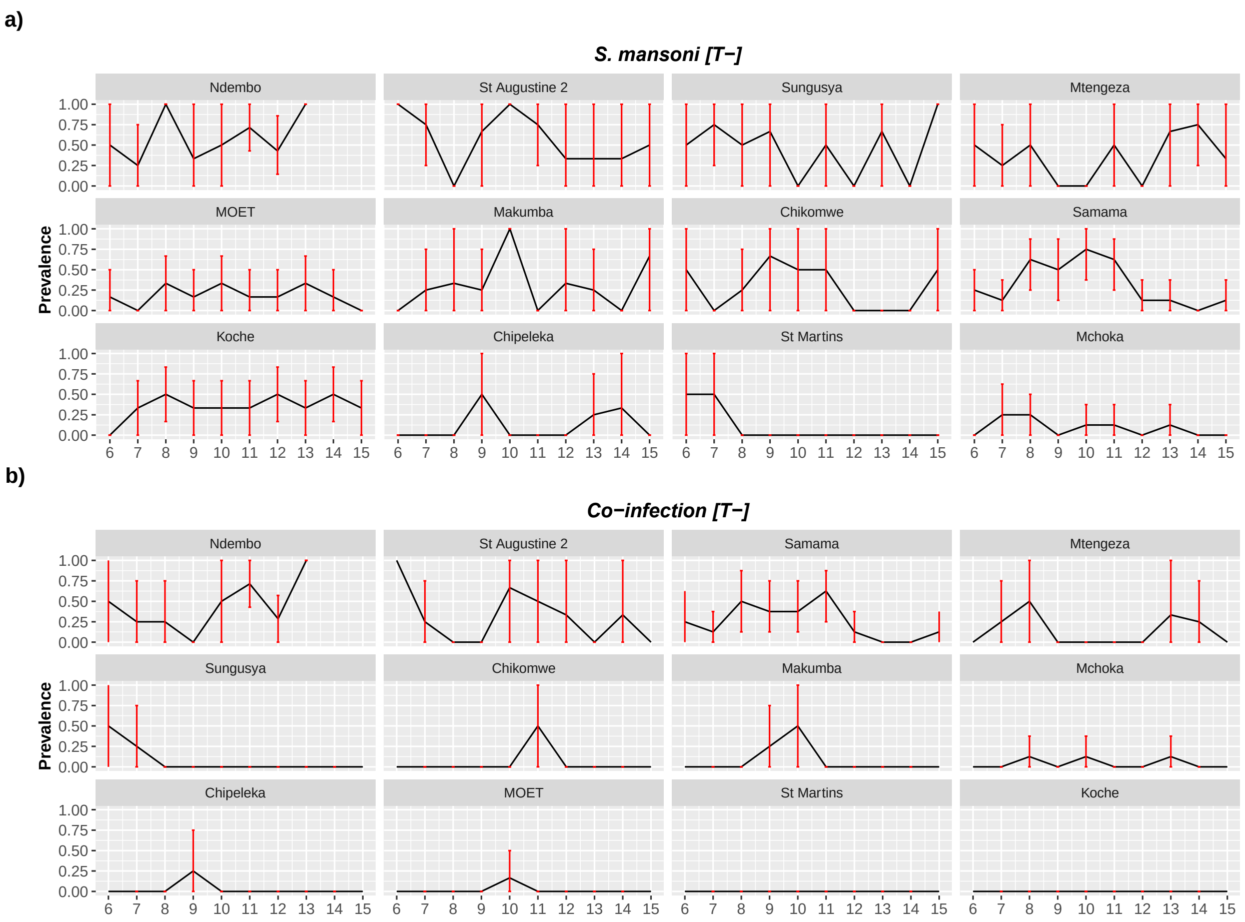

Figure A.2: Raw data plot showing the age of the children vs school prevalence for a) *S. mansoni* [T-], b) co-infection [T-] and c) Order of schools on heatmap was by highest to lowest prevalence.

B. Data methods

In brief, the GAMs in our secondary analysis took the form of a logistic regression using a Bernoulli distribution with mean probability p_{ij}. Let Y_{ij} be the diagnostic binary response for individual SAC i at a named school j. Two cases were considered: For dual-infection focus, Y_{ij} is either $Y_{ij} = 1$ if the SAC had a positive result for *S. haematobium* or *S. mansoni* or $Y_{ij} = 0$ if the SAC had a negative result for *S. haematobium* or *S. mansoni* at named school j. For co-infection focus: This follows that Y_{ij} is either $Y_{ij} = 1$ if the SAC had a positive result for both *S. haematobium* and *S. mansoni* or $Y_{ij} = 0$ if the SAC had at least one negative result. The GAM model takes the following form:

$$logit(p_{ij}) = log\left(\frac{p_{ij}}{1-p_{ij}}\right) = \alpha + \beta_i + s(age_{ij}; k),$$

with intercept α, β_i is vector of each school location with ith subject $i = 1,2, \dots, n$. s is a thin-plate spline function for age_{ij}, where age_{ij} denotes the age of the child i at school j, and k denotes the number of knots (estimated from the data). In all our analysis, the level of significance was set as ‘indication of ‘significance’ ˙p<0.1 or ‘significant; *p<0.05, ** p<0.01 or ‘highly significant’ ***p<0.001 and 95% confidence intervals were calculated for each model.

C. Prevalence heatmaps

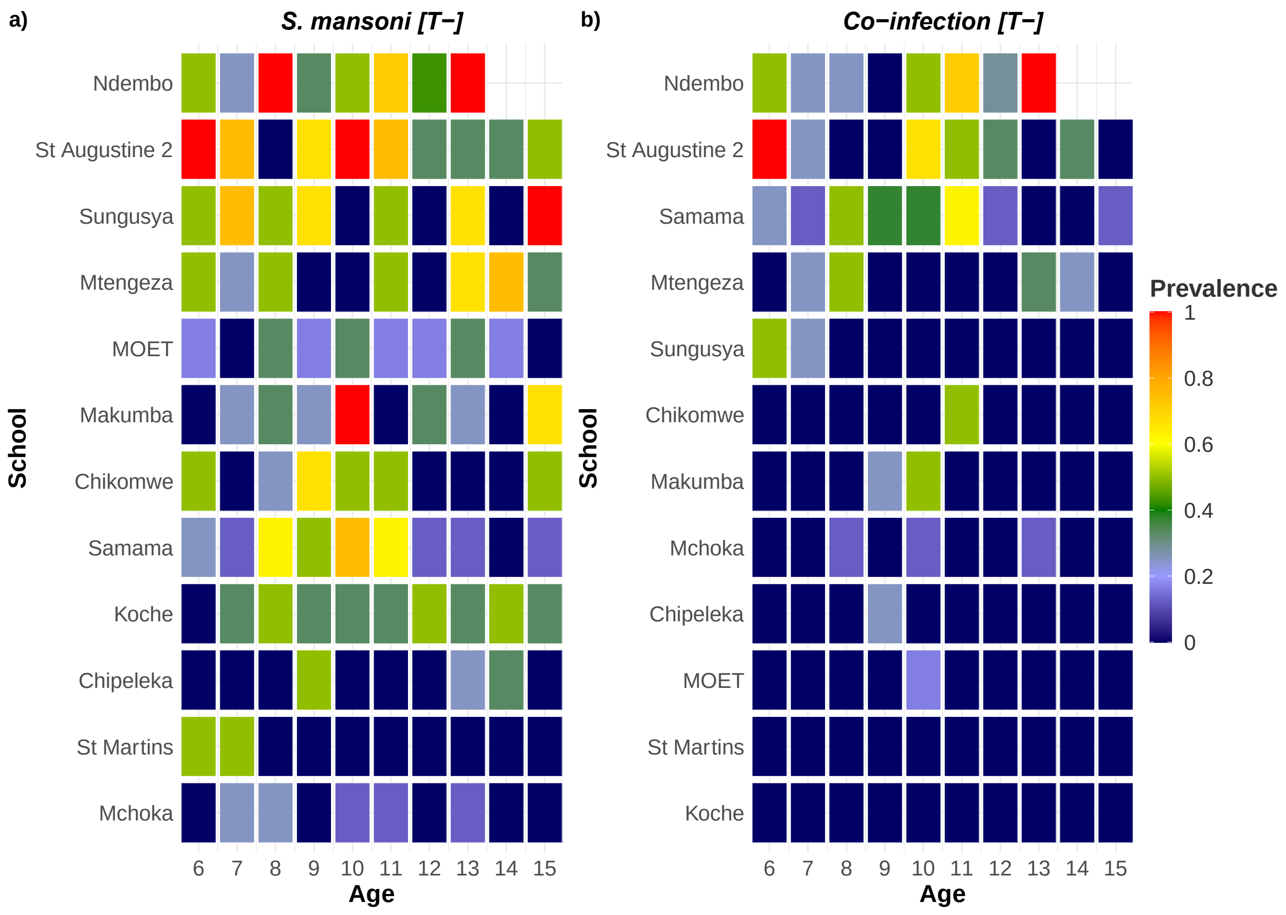

Figure C.1: Heatmap showing the age of the children vs school prevalence for a) *S. mansoni* [T-] and b) co-infection [T-]. Order of schools on heatmap was by highest to lowest prevalence.

D. Generalised additive models

Table D.1: Summary of prevalence of *S. mansoni* [T-] and co-infection [T-]

	S. mansoni (CCA) [T-]		Co-infection [T-]	
Name	**No. Positive (%)**	**95% CI**	**No. Positive (%)**	**95% CI**
Mchoka (N=80)	7 (8.75)	2.50-15.0	3 (3.75)	0.00-8.75
Samama (N=80)	26 (32.5)	22.5-42.5	20 (25.0)	16.3-35.0

MOET (N=60)	11 (18.3)	9.96-28.3	1 (1.67)	0.00-5.00
Koche (N=60)	21 (35.0)	23.3-46.7	0 (0.00)	-
St Augustine 2 (N=30)	17 (56.7)	40.0-73.3	9 (30.0)	13.3-46.7
Ndembo (N=30)	17 (56.7)	40.0-73.3	12 (40.0)	23.3-56.7
Sungusya (N=30)	14 (46.7)	30.0-63.3	2 (6.67)	0.00-16.7
St Martins (N=30)	3 (10.0)	0.00-20.0	0 (0.00)	-
Chikomwe (N=30)	8 (26.7)	13.3-43.3	2 (6.67)	0.00-16.7
Chipeleka (N=30)	4 (13.3)	3.33-26.7	1 (3.33)	0.00-10.0
Makumba (N=30)	9 (30.0)	13.3-46.7	2 (6.67)	0.00-16.7
Mtengeza (N=30)	10 (33.3)	16.7-50.0	4 (13.3)	3.33-26.6
Total (N=520)	147 (28.3)	24.4-32.1	56 (10.8)	8.08-13.5

Table D.2: GAM with smooth term age adjusted for school

	***S. mansoni* [T-]**		**Co-infection [T-]**	
		95% Cl		95% CI
**Smooth term (*p-value*)**				
**Age**	0.111		32.0e-2*	
Factor (esti-mated *coeffi-cient)*				
School				
Samama	1.63***	(0.718, 2.54)	2.21**	(0.939, 3.48)
MOET	0.856$^{\cdot}$	(-0.162, 1.87)	-0.838	(-3.13, 1.46)
Koche	1.74***	(0.797, 2.68)	-28.3	(-178, 178)
St Augustine 2	2.61***	(1.55, 3.68)	2.43***	(1.01, 3.84)
Ndembo	2.53***	(1.47, 3.60)	2.65***	(1.27, 4.03)
Sungusya	2.21***	(1.16, 3.28)	0.618	(-1.24, 2.47)
St Martins	0.132	(-1.29, 1.56)	-28.3	(-254, 254)
Chikomwe	1.33	(0.201, 2.45)	0.611	(-1.24, 2.46)
Chikomwe	0.461	(-0.851, 1.77)	-0.146	(-2.46, 2.17)
Makumba	1.51**	(0.408, 2.62)	0.681	(-1.17, 2.54)

Mtengza	1.67**	(0.580, 2.76)	1.45˙	(-0.133, 3.03)
Mchoka	0	0	0	0

Significance p<0.05, **Significance p<0.01, ***Significance p<0.001, ˙ Significance at p<0.1

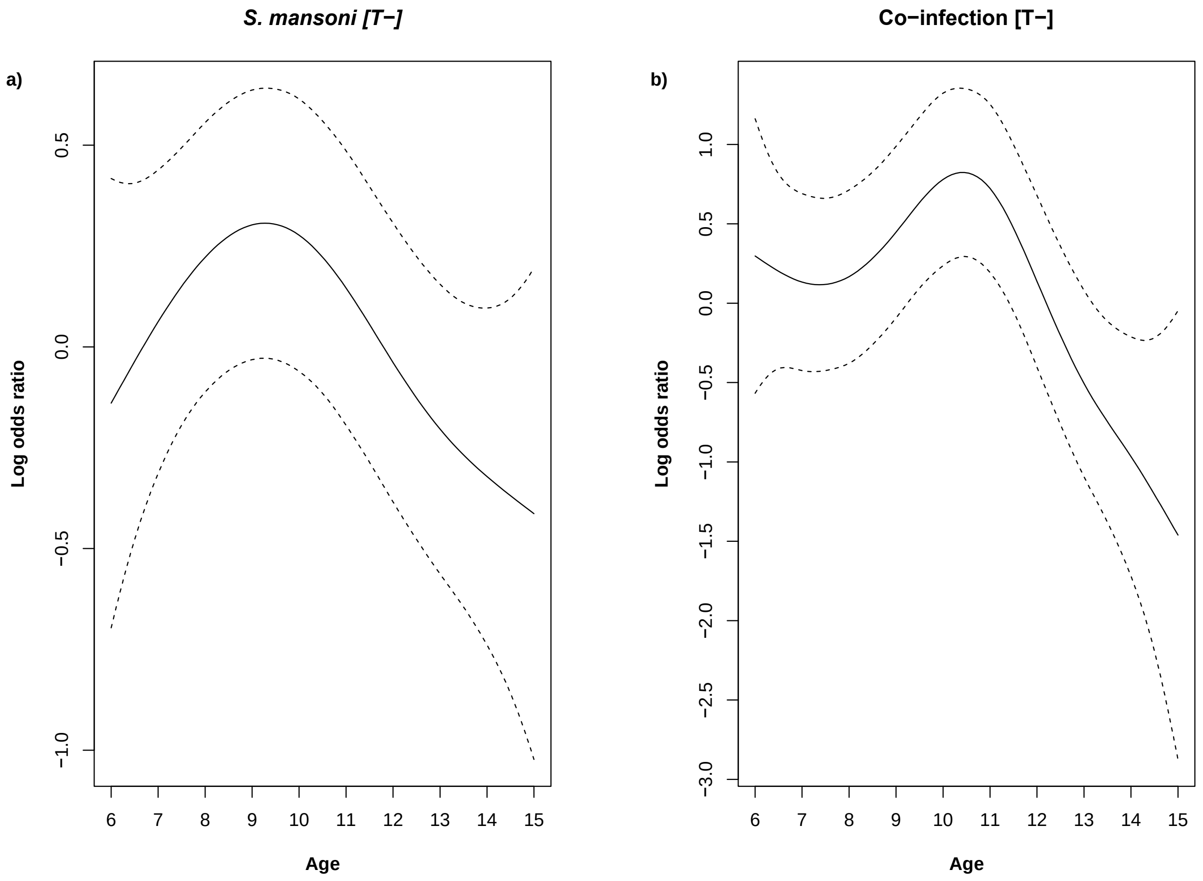

Figure D.1: Smooth age term plot for the GAM of *Schistosoma* association with age of SAC for a) *S. mansoni* [T-] and b) co-infection [T-].

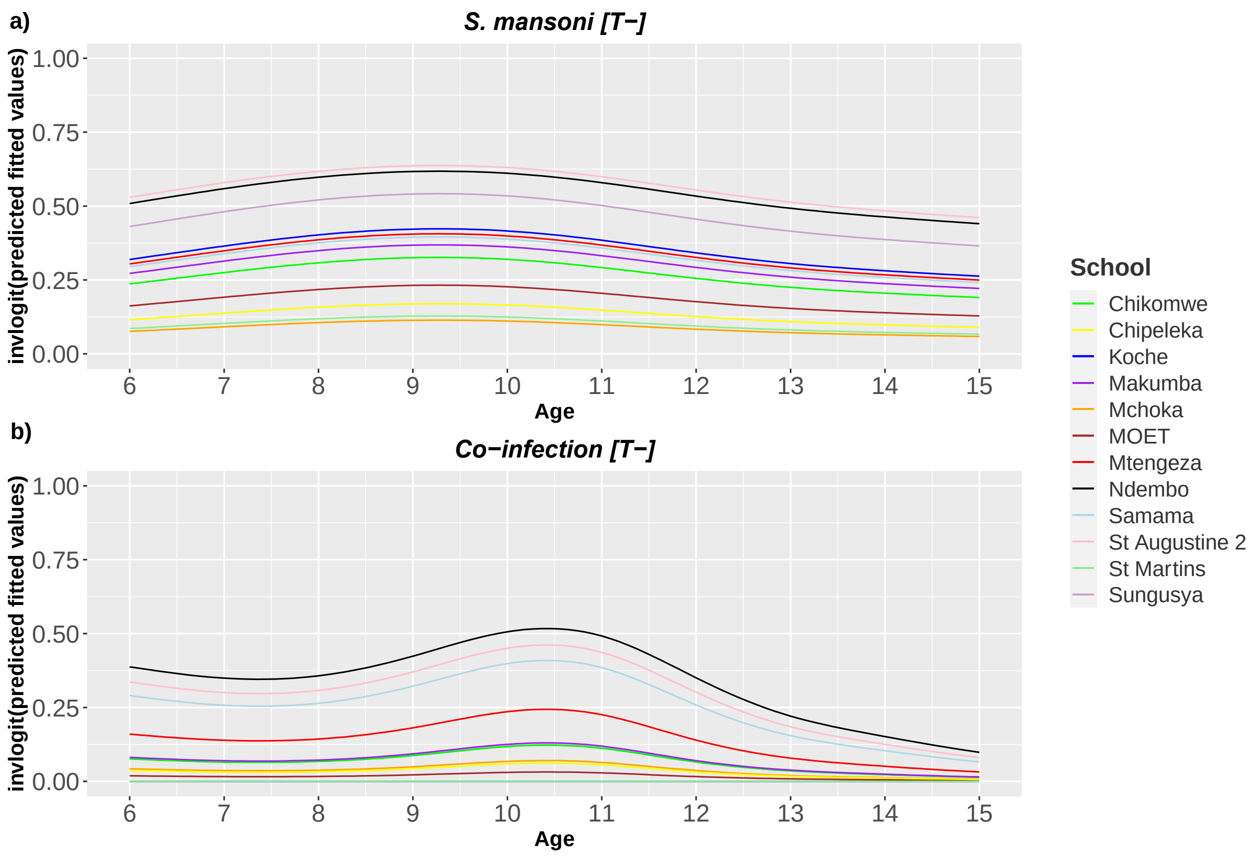

Figure D.2: Gam of *Schistosoma* association with age of SAC for each school. Invlogit of predicted fitted values versus age, a) *S. mansoni* [T-] and b) co-infection. *Light Green: Chikomwe, Yellow: Chipelekera, Dark Blue: Koche, Purple: Makumba, Orange: Mchoka, Brown: Moet Red: Mtengeza Black: Ndembo, Light Blue: Samama , Pink: St Augustine 2, Dark Green: St Martins, Mauve: Sun-gusya*

E. Residuals of GAMs

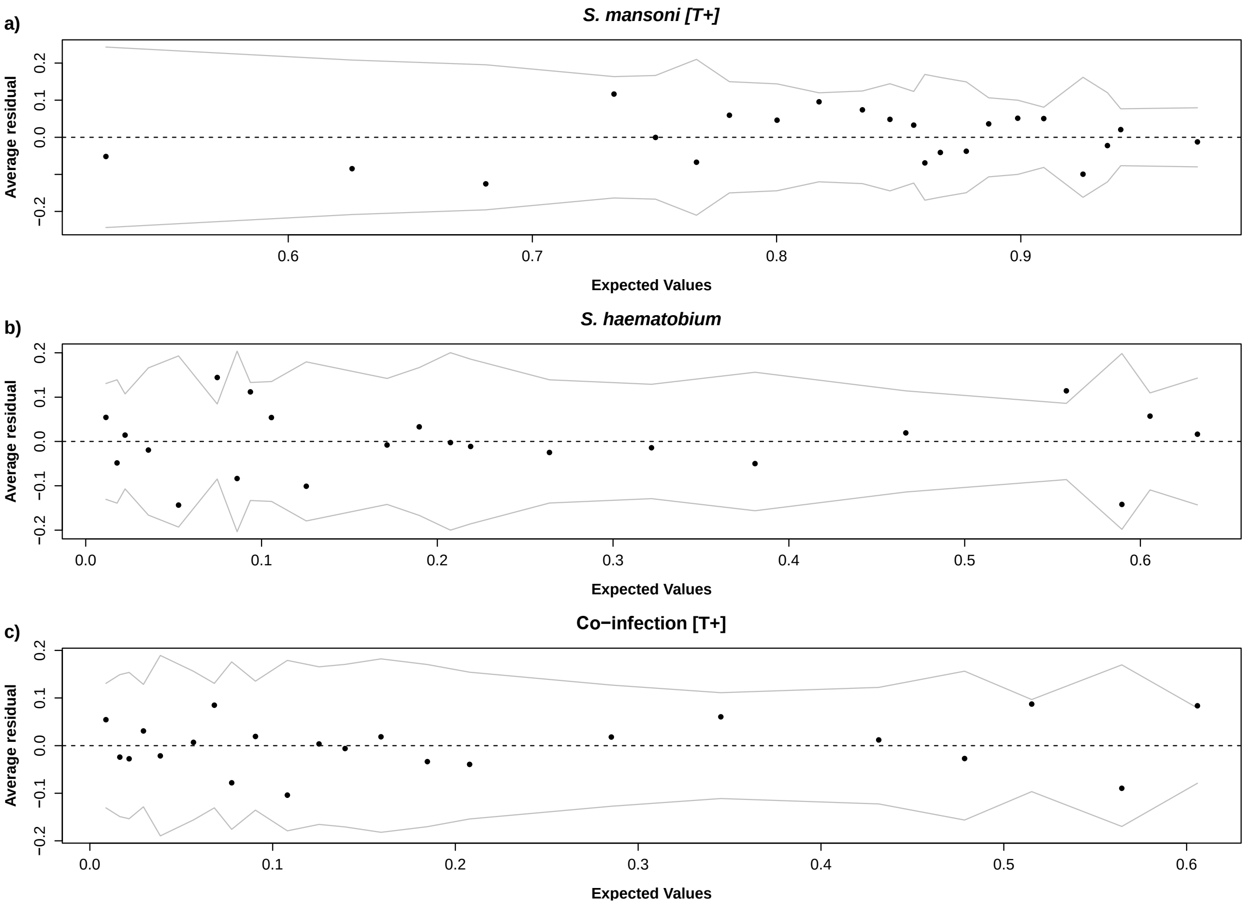

Figure E.1: Probability of the being positive with *Schistosoma* versus the average residuals, a) *S. mansoni* [T+], b) *S. haematobium* and c) co-infection [T+].

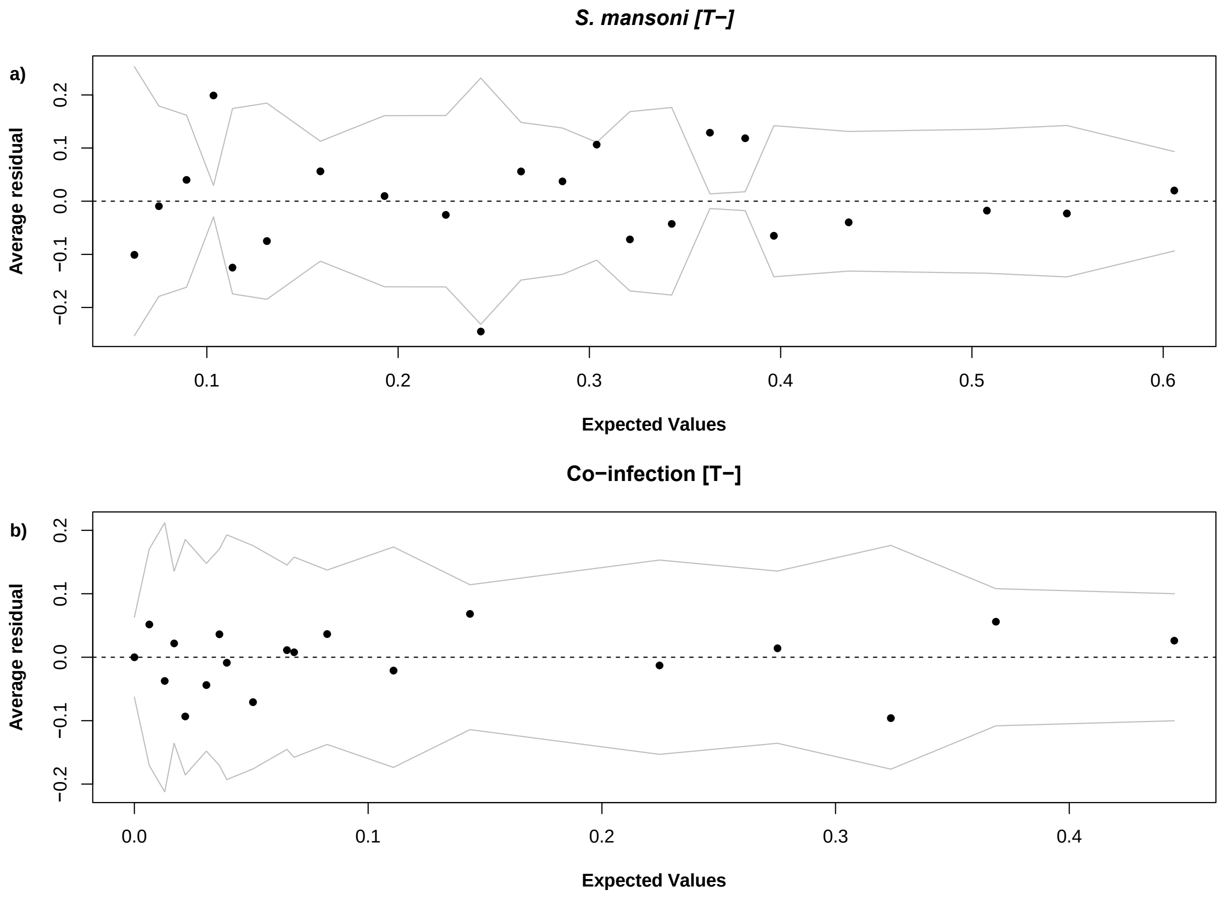

Figure E.2: Probability of the being positive with *Schistosoma* versus the average residuals a) *S. mansoni* [T-] and b) co-infection [T-].
